# Allergen Sensitivity Patterns and Their Correlation With Total Serum IgE Levels and Absolute Eosinophil Counts Among Patients With Allergic Rhinitis and Asthma in North Karnataka

**DOI:** 10.7759/cureus.67183

**Published:** 2024-08-19

**Authors:** Pranavi V, Keertivardhan D Kulkarni

**Affiliations:** 1 Respiratory Medicine, Tagore Medical College and Hospital, Chennai, IND; 2 Respiratory Medicine, Shri B M Patil Medical College, Hospital and Research Center, Bijapur Lingayat Development Education (BLDE) (Deemed to be University), Vijayapura, IND

**Keywords:** allergic rhinitis (ar), eosinophil counts, total ige, allergen sensitivity, skin prick test

## Abstract

Introduction

Respiratory allergies are prevalent across all populations and age groups, with the specific types of allergens varying according to geographic area, climate, location, economic status, and ethnic identity. While skin prick testing is considered the gold standard for assessing specific IgE against particular allergens, several factors can make the test less preferred. Consequently, total serum IgE levels and eosinophil counts are often used instead.

Objectives

The study aimed to determine allergen sensitivity patterns among patients with allergic rhinitis and/or asthma and to correlate skin prick test (SPT) reactivity with total serum IgE levels and absolute eosinophil count (AECs). This was done to assess the potential use of these measures as screening tests.

Materials and methods

A cross-sectional study was conducted on 44 patients diagnosed with allergic rhinitis and/or asthma. Each patient underwent an allergen SPT, and measurements were taken for total serum IgE levels and eosinophil counts. The study identified the most common allergens resulting in positive SPTs. Pearson correlation test was used for continuous variables, and a p-value of <0.05 was considered statistically significant.

Results

The study found that the majority of patients had only allergic rhinitis (17, 38.6%), followed by those with only asthma (12, 27.3%), and those with both allergic rhinitis and asthma (15, 34.1%). The most common allergen was Blomia (house dust mite), affecting 22 (50%) patients, while the least common were honey bee and lemon, each affecting 1 (2.3%) patient. There was a significant correlation between total serum IgE levels and AECs (p < 0.05). Additionally, a statistically significant correlation was found between total serum IgE levels, eosinophil counts, and the number of allergens to which patients were sensitized.

Conclusion

Blomia (house dust mite) is the most common allergen among patients with respiratory allergies in North Karnataka. While total serum IgE levels and AECs may help identify the extent of allergen sensitivity, the SPT remains the gold standard.

## Introduction

Respiratory allergies are the most common allergies among various populations and age groups worldwide, making them a significant health problem. In both developed and developing countries, the prevalence of allergic diseases is steadily increasing, ranging from 15% to 30% globally [1,2]. Allergic rhinitis constitutes more than 50% of all allergies seen in India [3], and more than 15 million people in the country are affected by asthma [4].

The types of allergens vary according to geographic area, climate, location, economic status, ethnic identity, and other factors. India, being a populous country with different climatic conditions and diverse food habits, exhibits a pattern of allergen sensitivity that varies from place to place [5].

Skin prick testing remains the gold standard for assessing specific IgE against a particular offending allergen [2,6]. However, several factors, such as higher costs, skin diseases, allergies, younger populations, and the risk of complications like anaphylaxis, make the test less preferred. Consequently, the detection of total serum IgE levels along with eosinophil counts is used to avoid these issues. In the past, total serum IgE was preferred as the screening test for diagnosing allergies by many physicians [2]. Peripheral blood eosinophil counts are also elevated in atopic individuals. Although these tests are not specific for a particular allergen, they are advantageous due to their lower cost and better patient compliance, as they are easy to perform. Various studies have shown a strong relationship between skin prick test (SPT) positivity, total serum IgE levels, and eosinophil count, but there is a scarcity of data among patients in the district of Vijayapura, Karnataka.

This study was undertaken to evaluate the allergen sensitivity patterns among patients in this region to facilitate better strategies for managing and preventing allergic rhinitis and extrinsic asthma. The goal was to target immunotherapy, reduce economic expenses, and improve the quality of life. The study also aimed to examine the correlation between total serum IgE levels and eosinophil counts with the SPT to determine the feasibility of using these measures as a screening test.

## Materials and methods

This cross-sectional study was conducted at the outpatient and inpatient Department of Respiratory Medicine, of a tertiary care hospital and a teaching institute in India, during the years 2021-2022. After taking institutional ethical clearance with IEC number BLDE (DU)/IEC/no-09/2021 from Bijapur Lingayat Development Education (BLDE) (Deemed to be University), a total of 44 patients with allergic rhinitis and/or asthma were enrolled, comprising 20 (45.5%) males and 24 (54.5%) females, with a mean age of 39.63 years (range 18-60 years). The diagnosis of asthma and allergic rhinitis was made according to the Global Initiative for Asthma (GINA) Guidelines 2020 and the Allergic Rhinitis and its Impact on Asthma (ARIA) Guidelines 2019, respectively. Patients not on any antihistamines for seven days were included in the study. The exclusion criteria were patients with exacerbation of asthma, those suffering from associated pulmonary diseases including tuberculosis and chronic obstructive pulmonary disease (COPD), patients on anti-IgE and steroid therapy, individuals with dermographism, and pregnant or lactating women.

A hemogram, sputum analysis, and chest radiography were performed to rule out other diagnoses. The study was approved by the Institutional Ethical Committee, and written consent was obtained from each patient for participation. All enrolled patients underwent an SPT for 73 common aeroallergens. Total serum IgE levels (expressed in KU/L) were measured using the enzyme-linked fluorescent assay (ELFA) for all patients. High total serum IgE levels were defined as >150 KU/L. Eosinophil counts were measured using automated hematology analyzers, with high eosinophil counts defined as >500 cells per cubic millimeter.

SPT

The SPT was conducted using 73 different types of allergens, which included three types of mites, nine types of fungi, nine types of pollens, six types of dust, 15 types of epithelia, and 31 types of food allergens. Saline was used as a negative control, and histamine was used as a positive control. The wheal diameter was measured after 15-20 minutes and was considered positive if it was 3 mm greater than the negative control.

The study includes patients aged 18 to 60 suffering from allergic rhinitis and/or asthma, who have not taken antihistamines for seven days and are willing to give informed consent for inclusion and skin prick testing. It excludes patients with asthma exacerbations, other pulmonary diseases like tuberculosis and chronic obstructive pulmonary disease, those on anti-IgE and steroid therapy, patients with dermographism, pregnant and lactating women, and anyone unwilling to participate or provide written consent.

Statistical analysis

Data was entered into MS Excel (Microsoft Corporation, Redmond, Washington, United States) and analyzed using the IBM SPSS Statistics for Windows, Version 20 (Released 2011; IBM Corp., Armonk, New York, United States). Categorical variables were compared using the Chi-square test. Continuous variables were compared using the Pearson correlation tests. p < 0.05 was considered statistically significant. All statistical tests were performed two-tailed.

## Results

Out of the 44 patients in the study, 18 (40.9%) were in the age group of 46-60 years, with a mean age of 39.63 years. There were 24 females (54.5%) and 20 males (45.5%). In terms of medical conditions, 17 patients (38.6%) had only allergic rhinitis, 12 patients (27.3%) had only asthma, and 15 patients (34.1%) had both allergic rhinitis and asthma. The descriptive statistics for the study population are presented in Table 1.

**Table 1:** Descriptive statistics for study population

Variables	Numbers (n)	Percentage (%)
Age in completed years		
15-30	13	29.5
31-45	13	29.5
46-60	18	40.9
Gender		
Male	20	45.5
Female	24	54.5
Allergic condition		
Asthma only	12	27.3
Allergic rhinitis only	17	38.6
Both allergic rhinitis and asthma	15	34.1
Family history of the study participants		
Yes	17	38.7
No	27	61.3
Body mass index (BMI) in kg/m2		
<18.5 (Underweight)	4	9.1
18.5 to 22.9 (ideal weight)	22	50
23.0 to 29.9 (overweight)	13	29.5
>30 (obese)	5	11.4
Type of diet consumed by the study participants		
Vegetarian	19	43.2
Nonvegetarian	25	56.8

The patients enrolled underwent SPT with 73 allergen extracts. SPT sensitivity pattern of patients with allergic rhinitis and/or asthma is shown in Table 2. 

**Table 2:** Skin prick test sensitivity pattern of patients with allergic rhinitis and/or asthma

Allergens		Skin prick test positivity						Total	
		Allergic rhinitis only		Asthma only		Allergic rhinitis and asthma			
		No. of cases	% of cases	No. of cases	% of cases	No. of cases	% of cases	No. of cases	% of cases
Mites	Dermatophagoides farinae	7	41.1	5	41.6	8	53.3	20	45.5
	Dermatophagoides pteronyssinus	6	35.2	6	50	6	40	18	40.9
	Blomia tropicalis	6	35.2	6	50	10	66.6	22	50
Fungi	Aspergillus fumigatus	1	5.8	2	16.6	1	6.6	4	9.1
	Aspergillus niger	1	5.8	1	8.3	3	20	5	11.4
	Aspergillus flavus	1	5.8	2	16.6	3	20	6	13.6
	Fusarium solani	3	17.6	2	16.6	1	6.6	6	13.6
	Rhizopus nigricans	2	11.7	2	16.6	1	6.6	5	11.4
	Curvularia lunata	2	11.7	2	16.6	2	13.3	6	13.6
	Cladosporium herbarum	2	11.7	3	25	1	6.6	6	13.6
	Candida albicans	1	5.8	3	25	2	13.3	6	13.6
	Trichoderma viride	1	5.8	1	8.3	2	13.3	4	9.1
Pollens	Cynodon dactylon	1	5.8	2	16.6	5	33.3	8	18.2
	Parthenium hysterophorus	2	11.7	1	8.3	4	26.6	7	15.9
	Xanthium strumarium	2	11.7	1	8.3	2	13.3	5	11.4
	Ageratum conyzoides	0	0	1	8.3	1	6.6	2	4.5
	Ricinus communis	3	17.6	2	16.6	1	6.6	6	13.6
	Zea mays	1	5.8	2	16.6	1	6.6	4	9.1
	Ipomoea batatas	2	11.7	1	8.3	1	6.6	4	9.1
	Cocos nucifera	1	5.8	1	8.3	0	0	2	4.5
	Peltophorum pterocarpum	4	23.5	1	8.3	0	0	5	11.4
Dust	Wheat dust	2	11.7	2	16.6	4	26.6	8	18.2
	Cotton dust	1	5.8	0	0	3	20	4	9.1
	House dust	0	0	2	16.6	3	20	5	11.4
	Paper dust	0	0	3	25	4	26.6	7	15.9
	Hay dust	1	5.8	1	8.3	2	13.3	4	9.1
	Grain dust (rice)	3	17.6	2	16.6	3	20	8	18.2
Epithelia	Dog epithelia	1	5.8	2	16.6	3	20	6	13.6
	Sheep's wool	0	0	1	8.3	2	13.3	3	6.8
	Human dander	1	5.8	1	8.3	1	6.6	3	6.8
	Chicken feather	1	5.8	2	16.6	0	0	3	6.8
	Ant (black)	0	0	1	8.3	2	13.3	3	6.8
	Cockroach	0	0	1	8.3	2	13.3	3	6.8
	Honey bee	0	0	1	8.3	0	0	1	2.3
	Cricket	1	5.8	1	8.3	2	13.3	4	9.1
	Mosquito	4	23.5	1	8.3	4	26.6	9	20.5
	House fly	2	11.7	1	8.3	3	20	6	13.6
	Rice weevil	1	5.8	1	8.3	0	0	2	4.5
	Moth	1	5.8	3	25	1	6.6	5	11.4
	Wasp	0	0	3	25	1	6.6	4	9.1
	Grasshopper	2	11.7	4	33.3	1	6.6	7	15.9
	Ant (red)	2	11.7	3	25	1	6.6	6	13.6
Foods	Wheat	1	5.8	1	8.3	1	6.6	3	6.8
	Bajra	2	11.7	1	8.3	2	13.3	5	11.4
	Jowar	2	11.7	1	8.3	1	6.6	4	9.1
	Gram Bengali	2	11.7	0	0	1	6.6	3	6.8
	Potato	2	11.7	0	0	1	6.6	3	6.8
	Maize	0	0	2	16.6	1	6.6	3	6.8
	Mustard	2	11.7	2	16.6	2	13.3	6	13.6
	Masoor dal	2	11.7	1	8.3	1	6.6	4	9.1
	Moong dal	3	17.6	0	0	0	0	3	6.8
	Dal arahar	3	17.6	0	0	1	6.6	4	9.1
	Tomato	2	11.7	2	16.6	1	6.6	5	11.4
	Milk	0	0	1	8.3	3	20	4	9.1
	Lemon	0	0	0	0	1	6.6	1	2.3
	Peanut	0	0	1	8.3	2	13.3	3	6.8
	Dal urad	2	11.7	4	33.3	1	6.6	7	15.9
	Mutton	1	5.8	2	16.6	0	0	3	6.8
	Chicken	1	5.8	1	8.3	3	20	5	11.4
	Gram kabuli	2	11.7	3	25	4	26.6	9	20.5
	Cashew nut	1	5.8	1	8.3	0	0	2	4.5
	Coriander	0	0	2	16.6	0	0	2	4.5
	Soya bean	1	5.8	1	8.3	0	0	2	4.5
	Fish sardine	0	0	1	8.3	2	13.3	3	6.8
	Egg white	1	5.8	1	8.3	1	6.6	3	6.8
	Brinjal	0	0	0	0	0	0	0	0
	Chocolate	1	5.8	0	0	1	6.6	2	4.5
	Coffee	0	0	2	16.6	1	6.6	3	6.8
	Coconut	1	5.8	0	0	1	6.6	2	4.5
	Gum acacia	1	5.8	1	8.3	1	6.6	3	6.8
	Curd	0	0	1	8.3	2	13.3	3	6.8
	Rajma	2	11.7	1	8.3	1	6.6	4	9.1
	Long pepper	1	5.8	0	0	1	6.6	2	4.5

The most common allergens identified were house dust mites, followed by food allergens, insects and epithelia, dust allergens, fungi, and pollens. Among individual allergens, the house dust mite (Blomia) was the most prevalent, with 22 (50%) showing sensitivity. The next most common allergens were mosquitoes and gram kabuli, each affecting nine (20.5%) patients. Sensitivity to *Cynodon dactylon* and wheat dust was observed in eight (18.2%) patients. The least common allergens, with only one (2.3%) patient showing sensitivity, were honey bee and lemon, while none of the patients were sensitive to brinjal. Among fungi, the most common allergens were *Aspergillus flavus, Fusarium solani,* *Curvularia lunata*, *Cladosporium herbarum*, and *Candida albicans*, each affecting six (13.6%) patients. *Aspergillus niger* and *Rhizopus nigricans *affected five (11.4%) patients each, while *Aspergillus fumigatus* and *Trichoderma* were the least common, with four (9.1%) patients each showing sensitivity. In terms of pollen allergens, sensitivity to* Cynodon dactylon *was the most prevalent, affecting eight (18.2%) patients, followed by *Parthenium hysterophorus* in seven (15.9%) patients. *Ricinus communis *affected six patients (13.6%), *Xanthium strumarium* and *Peltophorum pterocarpum* each affected five (11.4%) patients, *Zea mays* and *Ipomoea* each affected four patients (9.1%), and *Ageratum conyzoides* and *Cocos nucifera* each affected two (4.5%) patients. For dust allergens, wheat dust and grain dust (rice) were common, with eight (18.2%) patients showing sensitivity to each, followed by paper dust in seven (15.9%) patients, house dust in five (11.4%) patients, and cotton dust and hay dust in four (9.1%) patients. Overall, mosquitoes were the most common allergen among insects and epithelia, affecting nine (20.5%) patients, followed by grasshoppers in seven (15.9%) patients. The least common allergen in this category was honey bee, with only one (2.3%) patient showing sensitivity. Among food allergens, gram kabuli was the most common, affecting nine (20.5%) patients, followed by dal urad in seven (15.9%) patients, while none of the patients were sensitive to brinjal.

The study reported total serum IgE levels among patients with allergic rhinitis only, asthma only, and both allergic rhinitis and asthma. Among those with allergic rhinitis only, eight (47.1%) had IgE levels of ≤150 KU/L, while nine (52.9%) had levels of >150 KU/L. In the asthma-only group, all patients (12, 100%) had IgE levels of >150 KU/L. For those with both allergic rhinitis and asthma, six (40%) had IgE levels of ≤150 KU/L, and nine (60%) had levels of >150 KU/L as depicted in Table 3.

**Table 3:** Distribution of total serum IgE levels among the study patients

Total serum IgE	Allergic rhinitis only		Asthma only		Allergic rhinitis and asthma	
	No. of cases	% of cases	No. of cases	% of cases	No. of cases	% of cases
≤150 KU/L	8	47.1	0	0	6	40
>150 KU/L	9	52.9	12	100	9	60
Total	17	100	12	100	15	100

When we assesed their absolute eosinophil count (AEC), we found that in the allergic rhinitis only group, nine (52.9%) had AEC of <500 cells/cu.mm, while eight (47.1%) had AEC of >500 cells/cu.mm. Among those with asthma only, seven (58.3%) had AEC of <500 cells/cu.mm, and five (41.7%) had AEC of >500 cells/cu.mm. For patients with both allergic rhinitis and asthma, 10 (66.7%) had AEC of <500 cells/cu.mm, and five (33.3%) had AEC of >500 cells/cu.mm.as shown in Table 4. 

**Table 4:** Distribution of cases with eosinophil counts

Absolute eosinophil count (AEC)	Allergic rhinitis only		Asthma only		Allergic rhinitis and asthma	
	No. of cases	% of cases	No. of cases	% of cases	No. of cases	% of cases
<500 cells/cu.mm	9	52.9	7	58.3	10	66.7
>500 cells/cu.mm	8	47.1	5	41.7	5	33.3
Total	17	100	12	100	15	100

Table 5 illustrates the correlation between total serum IgE levels and AEC. Among individuals with total serum IgE levels ≤150 KU/L, 13(92.9%) had an AEC ≤500 cells/cu.mm, and 1(7.1%) had an AEC >500 cells/cu.mm. Among those with IgE levels >150 KU/L, 13(43.3%) had an AEC ≤500 cells/cu.mm, and 17(56.7%) had an AEC >500 cells/cu.mm. Overall, 26(59.1%) of the total cases had an AEC ≤500 cells/cu.mm, while 18(40.9%) had an AEC >500 cells/cu.mm. The Pearson correlation coefficient between total serum IgE levels and AEC was 0.733, indicating a strong positive correlation. This correlation is statistically significant, with a p-value of 0.00001. 

**Table 5:** Correlation between total serum IgE levels and absolute eosinophil counts *Statistically significant

Total serum IgE (KU/L)		Absolute eosinophil count (AEC)		Total	Pearson correlation coefficient	p-value
		≤500	>500			
≤150	Count	13	1	14	0.733	0.00001*
	% within IgE	92.9%	7.1%	100.0%		
>150	Count	13	17	30		
	% within IgE	43.3%	56.7%	100.0%		
Total	Count	26	18	44		
	% within IgE	59.1%	40.9%	100.0%		

Table 6 presents the correlation between total serum IgE levels and the number of allergens patients were sensitized to, as determined by a SPT. Among individuals with total serum IgE levels of ≤150 KU/L, 12 (85.7%) were sensitized to 0-10 allergens, and two (14.3%) were sensitized to 11-20 allergens. None were sensitized to more than 20 allergens. In the group with IgE levels of >150 KU/L, 20 (66.7%) were sensitized to 0-10 allergens, seven (23.3%) to 11-20 allergens, and 10% to 21-30 allergens. Overall, 32 (72.7%) of the total cases were sensitized to 0-10 allergens, nine (20.5%) to 11-20 allergens, and three (6.81%) to 21-30 allergens. The Pearson correlation coefficient between total serum IgE levels and the number of allergens sensitized to is 0.5121, indicating a moderate positive correlation. This correlation is statistically significant, with a p-value of 0.00038. 

**Table 6:** Correlation between total serum IgE levels and skin prick test positivity to allergens *Statistically significant

Total serum IgE levels (KU/L)		No. of allergens patients were sensitised to			Total	Pearson correlation coefficient	p-value
		0-10	11-20	21-30			
≤150	Count	12	2	0	14	0.5121	0.00038*
	% within IgE	85.7%	14.3%	0	100.0%		
>150	Count	20	7	3	30		
	% within IgE	66.7%	23.3%	10%	100.0%		
Total	Count	32	9	3	44		
	% within IgE	72.7%	20.5%	6.81%	100.0%		

Table 7 presents the correlation between AECs and SPT positivity to allergens. For patients with AEC ≤ 500 cells/cu.mm, 21 (80.8%) were sensitized to 0-10 allergens, five (19.2%) were sensitized to 11-20 allergens, and none were sensitized to 21-30 allergens. In the group with AEC > 500 cells/cu.mm, 11 (61.1%) were sensitized to 0-10 allergens, four (22.2%) to 11-20 allergens, and three (16.7%) to 21-30 allergens. Overall, 32 (72.7%) of the total cases were sensitized to 0-10 allergens, 9(20.5%) to 11-20 allergens, and three (6.81%) to 21-30 allergens. The Pearson correlation coefficient between AEC and the number of allergens sensitized to is 0.3078, indicating a weak positive correlation. This correlation is statistically significant, with a p-value of 0.042095. 

**Table 7:** Correlation between absolute eosinophil counts and skin prick test positivity to allergens *Statistically significant

Absolute eosinophil count (AEC)		No. of allergens patients were sensitized to			Total	Pearson correlation coefficient	p-value
		0-10	11-20	21-30			
≤500	Count	21	5	0	26	0.3078	0.042095*
	% within IgE	80.8%	19.2%	0	100.0%		
>500	Count	11	4	3	18		
	% within IgE	61.1%	22.2%	16.7%	100.0%		
Total	Count	32	9	3	44		
	% within IgE	72.7%	20.5%	6.81%	100.0%		

## Discussion

In patients with respiratory allergies, allergens are the primary triggers of symptoms. Identifying the most prevalent allergens in specific geographic areas is crucial for effective management, and numerous studies worldwide have focused on this. Allergen avoidance is a key component of management, and specific immunotherapy can be tailored based on the prevalent allergens in each area.

In the present study, there was a notable gender inclination toward females, which could be attributed to hormonal variations, and environmental or genetic factors. Among the patients, 12 (27.3%) had only asthma, 17 (38.6%) had allergic rhinitis alone, and 15 (34.1%) had both allergic rhinitis and asthma. These results are similar to a study conducted in Rajasthan, India, by Sharma et al., where patients with only allergic rhinitis were predominant (44.78%), followed by those with both allergic rhinitis and asthma (29.1%), and patients with only asthma were the least in number (26.12%) although the percentages of distribution differed [7]. A study done in Southwestern Iran by Moghtaderi et al. also showed a similar distribution [8]. Hence, family history of asthma and allergies is a strong determinant of allergic diseases.

Most patients in our study were nonvegetarians (56.8%). Both vegetarians and nonvegetarians were predominantly sensitized to food allergens followed by mites. Vegetarians were least sensitized to fungi (31.5%), while nonvegetarians showed the least SPT positivity toward pollens (40%).

House dust mites were the most common allergens that patients were sensitized to. This finding is consistent with other studies conducted in India. For instance, a study by Chogtu et al. identified house dust mites as the most common allergen causing nasobronchial allergy in 24.21% of patients [9]. Other regional studies reported sensitization rates of 77.13% in Mumbai, 60% in Allahabad, and 70% in North Kerala [10-12]. Among the house dust mites, Blomia was the most common offending allergen, particularly among patients with only asthma and those with both allergic rhinitis and asthma, whereas *Dermatophagoides farinae* was more common among those with only allergic rhinitis.

Among fungi, the common allergens identified were* Aspergillus flavus*, *Fusarium solani*, *Curvularia lunata*, *Cladosporium herbarum*, and *Candida albicans*, with 13.6% of patients showing SPT positivity for each. These results align with a study by Chogtu et al., where *Aspergillus flavus* was the most common allergen [9]. However, a study in Punjab by Jerath et al. reported *Cladosporium herbarum *as the most common allergen [13]. Several other studies have identified *Aspergillus fumigatus* as the most common allergen. In our study, *Fusarium solani* was the most prevalent among patients with allergic rhinitis only (17.6%). *Cladosporium herbarum* and *Candida albicans* were common among patients with asthma only (25%). *Aspergillus niger* and *Aspergillus flavus* were the common allergens among patients with both allergic rhinitis and asthma, found in 20% of the patients. These results are consistent with a study by Kunoor et al. in Central Kerala, which also reported *Aspergillus flavus* as the most common allergen among patients with both conditions [14].

Among pollens, sensitivity to *Cyanodon dactylon* was commonly observed in 18.2% of patients. Kumar et al. in Delhi also reported *Cyanodon dactylon* as the most common allergen [15]. In West Bengal, *Cocos nucifera *was the most common pollen causing sensitization. The variation in pollen sensitization across regions can be attributed to different climatic conditions. *Cyanodon dactylon* was common among patients with asthma and those with both allergic rhinitis and asthma, while *Peltophorum pterocarpum* was more common among patients with allergic rhinitis only.

Wheat dust and grain dust (rice) were the most common dust allergens. Grain dust (rice) was the most common allergen in patients with allergic rhinitis only, while paper dust was the most common among asthma-only patients. Wheat dust and paper dust were common among patients with both allergic rhinitis and asthma. A clinical profile study of allergic rhinitis in Central India also showed that grain dust (rice) was the common allergen with positive SPT results [16]. A study from Kerala also reported grain dust (rice) to be common among those with only asthma, which contradicts our findings [14].

Among insects and epithelia, mosquito was the most common allergen, with 20.5% of patients showing sensitivity. Other studies have reported varying results, such as cockroach being the most offending allergen in Uttar Pradesh and moth in Gujarat [17,18]. Mosquito was the common allergen among patients with allergic rhinitis and those with both conditions, while grasshopper was most common among asthma-only patients.

Regarding food allergens, 20.5% of patients showed sensitivity to Gram kabuli, the most common allergen. Among patients with allergic rhinitis only, Moong dal and Dal arahar were the most common allergens, while Dal urad was the most common among asthma-only patients. Gram kabuli was the most common allergen among patients with both conditions. Sensitivity to Moong dal was observed in patients with allergic rhinitis only, whereas sensitivity to coriander was found in asthma-only patients. Jain et al. reported milk as a significant trigger for allergic rhinitis patients, and Kumar et al. found maximum intolerance to cow milk in patients with bronchial asthma in Delhi [15,16]. The varying results of food allergen sensitivity in different regions might be due to differing dietary habits, highlighting the need for more studies on food allergen sensitivity patterns.

All asthma-only patients had elevated total serum IgE levels, consistent with Rasheed et al.'s report, where the majority of patients with both conditions had elevated total serum IgE levels (63.1%) [2]. Total serum IgE levels among atopic individuals correlate with the size of the target organ, being lowest in rhinitis, intermediate in asthmatics, and highest in atopic eczema, supporting our findings [19]. Out of 44 patients, 26 (59%) had normal eosinophil counts, while 18 (41%) had elevated counts, differing from studies by Arastu et al. and Muddaiah et al., which found that the majority of patients had elevated eosinophil counts [20,21]. In our study, most patients with only allergic rhinitis had elevated eosinophil counts. The discrepancy might be because blood eosinophilia is associated with more severe asthma, increased exacerbation rates, and resistance to treatment.

Of the 30 patients with elevated total serum IgE levels, 17 had associated elevated AECs. A significant positive correlation (p < 0.05) was found between total serum IgE levels and AECs, consistent with the results of Altaii et al. and Muddaiah et al. [21]. Only two patients (4.5%) were monosensitized, while the rest were sensitive to at least two allergens. Most patients (66.7%) with elevated total serum IgE levels were sensitized to at least 10 allergens, and 61.1% of patients with elevated eosinophil counts were sensitized to fewer than 10 allergens. Polysensitization might result from genetic or environmental factors favoring the growth and vegetation of specific plant species with similar survival conditions.

There was a positive correlation between total serum IgE levels and SPT positivity, as well as between AECs and SPT positivity, which were both statistically significant (p < 0.05). This indicates that higher levels of total serum IgE and eosinophil counts correspond to the number of allergens the patients are sensitized to. Rasheed et al. found that 25.5% of patients were monosensitized, differing from our results, although a significant correlation between total serum IgE levels and SPT positivity was established.

The study provides valuable insights into allergen sensitivity patterns specific to the North Karnataka region, highlighting the prevalence of certain allergens like Blomia (house dust mite. The use of multiple diagnostic tools, including SPTs, total serum IgE levels, and eosinophil counts, allows for a comprehensive assessment of allergen sensitivity and correlation analysis. The study's sample size of 44 patients may limit the generalizability of the findings.

## Conclusions

The results of this study provide comprehensive knowledge of the locally prevalent allergens in North Karnataka among patients with allergic rhinitis and/or asthma. Overall, house dust mites, particularly Blomia, were identified as the most common offending allergens. The study underscores the importance of allergen sensitization, mediated by IgE and eosinophils, on the basis of allergic diseases. Elevated total serum IgE levels can serve as a diagnostic criterion, especially among asthmatics, and the likelihood of sensitization to multiple allergens increases with IgE levels above the normal range. However, despite the positive correlation between total serum IgE, eosinophil counts, and SPT positivity, relying solely on IgE as a screening tool is insufficient, as IgE can be elevated in several other conditions. Therefore, the SPT remains the gold standard for diagnosing specific allergens, guiding appropriate treatment measures such as avoidance and immunotherapy.
